# Identification of *Tenrec ecaudatus*, a Wild Mammal Introduced to Mayotte Island, as a Reservoir of the Newly Identified Human Pathogenic *Leptospira mayottensis*

**DOI:** 10.1371/journal.pntd.0004933

**Published:** 2016-08-30

**Authors:** Erwan Lagadec, Yann Gomard, Gildas Le Minter, Colette Cordonin, Eric Cardinale, Beza Ramasindrazana, Muriel Dietrich, Steven M Goodman, Pablo Tortosa, Koussay Dellagi

**Affiliations:** 1 Centre de Recherche et de Veille sur les maladies émergentes dans l’Océan Indien (CRVOI), Plateforme de Recherche CYROI, Sainte Clotilde, Reunion Island, France; 2 Université de La Réunion, UMR PIMIT « Processus Infectieux en Milieu Insulaire Tropical », Plateforme de Recherche CYROI, Sainte Clotilde, Reunion Island, France; 3 UMR CMAEE « Contrôle des Maladies Animales, Exotiques et Emergentes », Plateforme de recherche CYROI, Sainte Clotilde, Reunion Island, France; 4 Center for Viral Zoonoses, Department of Medical Virology, Faculty of Health Sciences, University of Pretoria, Pretoria, South Africa; 5 Field Museum of Natural History, Chicago, Illinois, United States of America; 6 Association Vahatra, Antananarivo, Madagascar; University of Tennessee, UNITED STATES

## Abstract

Leptospirosis is a bacterial zoonosis of major concern on tropical islands. Human populations on western Indian Ocean islands are strongly affected by the disease although each archipelago shows contrasting epidemiology. For instance, Mayotte, part of the Comoros Archipelago, differs from the other neighbouring islands by a high diversity of *Leptospira* species infecting humans that includes *Leptospira mayottensis*, a species thought to be unique to this island. Using bacterial culture, molecular detection and typing, the present study explored the wild and domestic local mammalian fauna for renal carriage of leptospires and addressed the genetic relationships of the infecting strains with local isolates obtained from acute human cases and with *Leptospira* strains hosted by mammal species endemic to nearby Madagascar. Tenrec (*Tenrec ecaudatus*, Family Tenrecidae), a terrestrial mammal introduced from Madagascar, is identified as a reservoir of *L*. *mayottensis*. All isolated *L*. *mayottensis* sequence types form a monophyletic clade that includes *Leptospira* strains infecting humans and tenrecs on Mayotte, as well as two other Malagasy endemic tenrecid species of the genus *Microgale*. The lower diversity of *L*. *mayottensis* in tenrecs from Mayotte, compared to that occurring in Madagascar, suggests that *L*. *mayottensis* has indeed a Malagasy origin. This study also showed that introduced rats (*Rattus rattus*) and dogs are probably the main reservoirs of *Leptospira borgpetersenii* and *Leptospira kirschneri*, both bacteria being prevalent in local clinical cases. Data emphasize the epidemiological link between the two neighbouring islands and the role of introduced small mammals in shaping the local epidemiology of leptospirosis.

## Author Summary

Islands are exceptionally prone to species introduction, including pathogens with detrimental public health consequences for which the invasive alien species could act as reservoirs. Our study shows how the local non-native mammal fauna of Mayotte Island is associated with the introduction and epidemiology of human leptospirosis, a zoonosis strongly affecting tropical islands. Data presented herein identify *Tenrec ecaudatus* (Family Tenrecidae), an omnivorous species introduced from neighbouring Madagascar, as a reservoir of the recently named *Leptospira mayottensis*. Further, we suggest a Malagasy origin of *L*. *mayottensis*, which occurs naturally within the Tenrecidae radiation endemic to Madagascar. Finally, we provide evidence that dogs and rats are reservoirs for other *Leptospira* lineages of medical importance on Mayotte. Altogether, our study highlights the impact of species introduction on human health and further suggests that the biogeography of microorganisms in insular ecosystems, including pathogenic endemic lineages, should be considered from evolutionary and medical perspectives.

## Introduction

Leptospirosis is a bacterial zoonosis caused by pathogenic spirochetes of the genus *Leptospira*. Human infection is common in tropical countries where warm and humid conditions favour the survival of *Leptospira* spp. in water and soil. Furthermore, human populations in these countries often live in rural areas or in impoverished urban zones with inadequate sanitation and can be at higher risk of exposure to the infection via the contaminated environment. A wide variety of mammal species can be infected by *Leptospira* spp. but not all act as reservoirs. Animal reservoirs support the chronic colonization of their renal tubules by a biofilm of leptospires and thus, release over prolonged periods the bacteria into the environment via their urine. Rodents and particularly rats, especially *Rattus* spp., are considered as the principal reservoir of these pathogenic bacteria and the main source of human infection, a role likely favoured by the worldwide distribution and commensal behaviour of some invasive species in this genus.

Two methods are currently used to identify leptospires: a phenotypic classification based on the microscopic agglutination test (MAT) that recognizes over 25 serogroups and 300 serovars [1] and a more recently introduced genetic classification based on bacterial DNA sequences that allows the identification of 20 *Leptospira* spp., nine of which are pathogenic [2]. Genotyping through Multiple Locus Sequence Typing (MLST) based on a selected set of housekeeping genes [3] has become a method of choice to identify *Leptospira* at the species and infra-species levels. Indeed, molecular typing allows deciphering the molecular epidemiology of the disease, sharing data among laboratories investigating distant geographic regions and analyzing the evolution of host-parasite relationships using robust molecular characters [4].

The incidence of human leptospirosis has been reported to be highest on tropical islands and this observation holds true for the southwestern Indian Ocean (SWIO) region [5]. Poorly documented on Mauritius and Madagascar, the incidence of the disease in the Seychelles ranks first worldwide among surveyed countries [5], while on Reunion Island, a French overseas department, the rate of human leptospirosis has been reported to be nearly 20 times higher than in continental France [6].

Mayotte, the most southern island of the Comoros Archipelago is a French overseas department of about 375 km², located in the northern entrance of the Mozambique Channel about 300 km off the northwestern coast of Madagascar. Human leptospirosis is highly prevalent on the island with an annual incidence in 2013 estimated at 35 per 100,000 [6]. Most interestingly, *Leptospira* strains isolated from patients, hereafter referred to clinical isolates, showed a much larger species diversity on Mayotte than on Reunion Island: 16 different sequence types (STs) were identified by MLST on Mayotte [7], while only three on Reunion Island [8]. Further, pathogenic leptospires causing acute human infections on Mayotte were identified as *Leptospira interrogans*, *Leptospira borgpetersenii*, *Leptospira kirschneri* and members of a previously undefined phylogroup that has recently been named as *Leptospira mayottensis* [7,9]. By contrast, only *L*. *interrogans* and *L*. *borgpetersenii* have been reported on Reunion Island [8]. The high diversity of leptospires on Mayotte, unique so far within the SWIO region, likely reflects local eco-epidemiologic specificities of animal reservoirs. With the exception of bats, no native small mammals occur on Mayotte [7]. Although one study has previously investigated animal reservoirs on Mayotte [7], it targeted *Rattus rattus*, the only rat species known on the island, for kidney carriage of pathogenic leptospires, whereas other species of the local wild and domestic fauna were screened by MAT, a serological test that provides evidence of previous infections but does not assess the actual carrier state of the investigated animals.

Through the investigation of the local wild and domestic fauna, the present study aimed at identifying animal reservoirs of leptospires on Mayotte, with a particular focus on *L*. *mayottensis*. This leptospiral species has been recently detected in wild small mammals endemic to neighbouring Madagascar [4] and, hence, it can be hypothesized that the geographic proximity and various socio-economic exchanges between these two islands could have facilitated the introduction of this pathogenic bacteria to Mayotte.

## Methods

### Ethics statement

All animal procedures carried out in this study were performed in accordance with the European Union legislation for the protection of animals used for scientific purposes (Directive 2010/63/EU). The ethical terms of the research protocol were approved by the CYROI Institutional Animal Care and Use Committee (Comité d’Ethique du CYROI n° 114, IACUC certified by the French Ministry of Higher Education and Research) under accreditation 03387 (LeptOI) and 03584 (BatMan).

### Animal sampling

Mammals were trapped on Mayotte during three field sessions carried out from July 2012 to December 2014. *Rattus rattus* and *Tenrec ecaudatus* were captured using live traps baited with grilled coconut at eight and five localities, respectively. Sampling sites were selected in order to maximize the species diversity and geographic distribution of mammals in our sample. Trapping localities included a variety of different ecological settings across the island (see S1 Fig). In a local laboratory, *R*. *rattus* were sacrificed by cervical dislocation and *T*. *ecaudatus* by percussive blow to the head. For each individual, tissue was quickly collected from kidney, lung and spleen, pooled together and immediately stored in dry vials at -80°C. Insectivorous bats, *Chaerephon* sp. (Family Molossidae), were captured at dusk or at night using mist nets placed either in the vicinity of synanthropic roost sites or across flyways, while frugivorous bats, *Pteropus seychellensis comorensis* (Family Pteropodidae), were captured at night using mist nets set around fruiting trees. Captured bats were individually placed in clean cloth bags and brought back to a local laboratory. In most cases, animals spontaneously urinated after their removal from holding bags allowing the collection of urine. Bats were subsequently released at dusk at the initial capture site. Dogs (*Canis lupus familiaris*) were only manipulated and sampled by a local veterinary doctor; kidneys were obtained from animals that were euthanized for medical purposes, when possible urine collected from these same animals and immediately kept in dry vials at -80°C. In addition, urine samples were obtained from non-euthanized dogs through aseptic urethral catheterization. Kidneys from zebus (*Bos indica*) were purchased at the slaughterhouse of Mayotte (Kaweni) or obtained during traditional butchering. All samples were sent in liquid nitrogen to Reunion Island for analyses.

### *Leptospira* spp. isolation

In addition to the sampling and storage at -80°C of organs and urine mentioned above, a few urine droplets and/or a small piece of freshly sampled kidney (crushed under sterile conditions) were used individually to inoculate three distinct culture media: (i) Ellinghausen-McCullough-Johnson-Harris (EMJH) liquid medium (Difco, Detroit, MI, USA) supplemented with Albumin Fatty Acid Supplement (AFAS; Royal Tropical Institute, Amsterdam, Netherlands) [10] (ii) EMJH liquid medium supplemented with AFAS, rabbit serum and fetal calf serum (1% each); and (iii) semisolid Fletcher medium (Difco, Detroit, MI, USA) supplemented with rabbit serum (8%). All media were supplemented with 5-fluorouracil (5-FU) at a final concentration of 200 μg.mL^-1^. Cultures were incubated at 28°C, visually checked for the presence of leptospires using a dark field microscope once a week for four months and positive cultures were further sub-cultured in fresh EMJH liquid medium deprived of 5-FU. DNA was extracted from 1 mL of each positive culture using the EZ1 Biorobot with Qiagen EZ1 DNA Tissue kits (Qiagen, Les Ulis, France).

### Detection of pathogenic leptospires

Approximately 1 mm³ of pooled organs (kidney, lung and spleen) from each *R*. *rattus* and *T*. *ecaudatus* specimen and only kidney from dogs and zebus were dissected on sterile ice and further processed as previously described [11]. Thirty microliters of urine from bats and dogs were combined with 120 μL of Dulbecco’s modified medium (GIBCO, Grand Island, NY, USA) and 50 μL of ATL buffer (Qiagen, Les Ulis, France). Subsequently, total nucleic acids were extracted from urine or homogenized tissues by using an EZ1 extraction robot and the EZ1 Virus Mini Kit version 2.0. A reverse transcription step was performed on total nucleic acids with GoScript Reverse Transcriptase (Promega, Madison, WI, USA) to obtain cDNA. Detection of a portion of the 16S rRNA gene of pathogenic *Leptospira* spp. was then carried out on 5 μL of cDNA using a probe-specific real-time Polymerase Chain Reaction system (RT-PCR) [12].

### *Leptospira* spp. genotyping

*Leptospira* spp. in samples testing positive by RT-PCR and/or culture were genotyped using a previously described MLST scheme encompassing six genes: *secY*, *adk*, *rrs2*, *icdA*, *lipL32* and *lipL41* [13], and recently optimized to improve the amplification of SWIO lineages [4]. RT-PCR is more sensitive than any MLST PCR (likely because of the very short length of RT-PCR amplicon), and some samples that were positive by the former detection system were MLST negative. In an attempt to further characterize *Leptospira* spp. from these particular samples, we used an alternative primer set (LA-LB) targeting a shorter piece of *rrs2* [14]. The amplification of each marker was realized with GoTaq Hot Start Green Master Mix 2X (Promega, Madison, WI, USA) and further sequenced on both strands by direct Sanger sequencing (Genoscreen, Lille, France) using the same amplification primer sets. All sequences were deposited on GenBank under the following accession numbers: KT338823-KT338942, KT725237-KT725243 and KX427207-KX427227.

### Phylogenetic analyses and genetic diversity

Accessible sequences from clinical *Leptospira* isolates from Mayotte were included in the study [15]. Since *icdA* sequences were not provided for these clinical isolates, we used five out of the six markers of the MLST scheme in our analyses. In addition, we added bacterial sequences obtained from endemic terrestrial small mammals from Madagascar, *Microgale cowani*, *Microgale dobsoni*, *Microgale majori*, *Microgale longicaudata* and *Microgale principula* (Family Tenrecidae, Subfamily Oryzorictinae). The complete MLST of these *Leptospira* strains were sequenced in a previous study [4] and from cultures realized during field missions carried out in the Central Highlands of Madagascar, Réserve Spéciale d’Ambohitantely, in March and October 2014 [16].

Phylogenetic analyses were performed on each gene separately and subsequently on concatenated sequences using the best model of sequence evolution determined by jModelTest v.0.1.1[17] for each dataset. Bayesian analyses were performed with MrBayes 3.1.2 [18] and consisted of two independent runs of four incrementally heated Metropolis Coupled Markov Chain Monte Carlo (MCMCMC) starting from a random tree. MCMCMC was run for 10,000,000 generations with trees and associated model parameters sampled every 100 generations. The convergence level of each phylogeny was validated by an average standard deviation of split frequencies inferior to 0.05. The initial 10% of trees from each run were discarded as burn-in and the consensus phylogeny along with posterior probabilities were obtained from the remaining trees. Bayesian trees were visualized and rooted to midpoint with FigTree v.1.3.1 (Andrew Rambaut, Institute of Evolutionary Biology, University of Edinburgh, 2006 to 2009; http://tree.bio.ed.ac.uk/).

Genetic diversity was compared between the identified clades in the multilocus phylogeny by estimating the nucleotide diversity (π) within each clade from the concatenated sequences using DNASP v.5.10.01[19].

## Results

Altogether, 486 samples of tissue and/or urine collected on Mayotte from 289 *Rattus rattus*, 37 *Tenrec ecaudatus*, 53 *Canis lupus familiaris*, 18 *Bos indica*, 69 *Chaerephon* sp. and 20 *Pteropus seychellensis comorensis* were screened for the presence of *Leptospira* spp. by RT-PCR and/or culture. The results are summarized on Table 1.

**Table 1:** Animals from the wild and domestic fauna of Mayotte tested for carriage of *Leptospira* spp. by real-time PCR, culture, and genotyping.

Host species	Number of animals	Number of animals	Number of *Leptospira*	Number of typed	*Leptospira* spp.			
	tested by RT-PCR	tested by culture	spp. positive animals	*Leptospira* strains				
	(OP, K, U)*	(K, U)*	(%)		*Lb*	*Lm*	*Li*	*Lk*
***Rattus rattus***	289	81	46 (15.9)	21	13	0	3	5
	(OP = 289)	(K = 81)	(38PCR+/Culture-					
			8PCR+/Culture+)					
***Tenrec***	37	36	10 (27.0)	8	0	8	0	0
***ecaudatus***	(OP = 37)	(K = 36, U = 17, 17†)	(2PCR+/Culture-					
			8PCR+/Culture+)					
***Bos indica***	18	0	1 PCR+ (5.6)	0	0	0	0	0
	(K = 18)							
***Canis lupus***	53	0	7 PCR+ (13.2)	6	3	0	0	3
***familiaris***	(K = 38, U = 45, 30†)							
***Chaerephon***	69	69	0	0	0	0	0	0
**sp.**	(U = 69)	(U = 69)						
***Pteropus***	20	20	2 PCR+ (10.0)	1	0	0	0	1
***seychellensis***	(U = 20)	(U = 20)						
***comorensis***								

*Lb*: *L*. *borgpetersenii*, *Lm*: *L*. *mayottensis*, *Li*: *L*. *interrogans*, *Lk*: *L*. *kirschneri*.

* OP: organ pool, K: kidney, U: urine.

† Number of animals with paired sampling (OP/K+U)

### *Leptospira* spp. detection by RT-PCR and culture

Pathogenic *Leptospira* spp. were detected by RT-PCR in 27.0% of *T*. *ecaudatus*, 15.9% of *R*. *rattus*, 13.2% of dogs, 10.0% of *P*. *seychellensis comorensis* and 5.6% of zebus. All urine samples from *Chaerephon* sp. tested negative by RT-PCR. Bacterial cultures were attempted using 117 freshly sampled kidney and 94 urine samples. Overall, leptospires culture was successful for samples testing positive through RT-PCR only. Among the 81 *R*. *rattus* tested by culture inoculated with kidney, 14 were positive by RT-PCR, of which eight allowed positive cultures (57.1%). Similarly, eight of the 10 RT-PCR-positive samples (80.0%) from *T*. *ecaudatus* allowed isolation of leptospires by culture. Two of them (MDI295 and MDI321) yielded positive cultures from both urine and kidney samples. Lastly, no isolate grew in urine cultures from RT-PCR positive *P*. *seychellensis comorensis* samples. No culture was attempted when sampling dogs or zebus.

### *Leptospira* species diversity in wild and domestic mammals

We were able to PCR amplify all six loci of the MLST scheme using DNA extracted from all 16 *Leptospira* cultures, obtained from eight *R*. *rattus* and eight *T*. *ecaudatus* samples. Interestingly, for *T*. *ecaudatus*, we failed to amplify the *lipL41* gene with the conventional primers from Ahmed *et al*.[13], and used alternative primers designed by Dietrich *et al*. [4]. As expected, PCR targeting MLST loci were more arduous when using DNA extracted from tissue and urine of PCR-positive samples for which culture had failed. For instance, no successful PCR targeting MLST loci was recorded for the two RT-PCR positive *T*. *ecaudatus* failing to yield positive culture. Using the MLST scheme, only *rrs2* locus was amplified from four PCR-positive *R*. *rattus*, while two *loci* (*secY* and *rrs2*) were amplified from two other *R*. *rattus*. Similarly, only *rrs2* was amplified from six out of the seven RT-PCR-positive dog samples and from one out of the two RT-PCR-positive *P*. *seychellensis comorensis* samples. Lastly, no sequence could be obtained from the single zebu sample testing positive by RT-PCR. In addition, for seven PCR-positive *R*. *rattus* samples failing to produce any data using the MLST scheme, we successfully amplified and sequenced the *rrs2* gene using an alternative primer set (LA-LB). Altogether, we could identify leptospiral diversity at the species level for 21 *R*. *rattus*, eight *T*. *ecaudatus*, six dogs and one frugivorous bat from Mayotte (see Table 1). *Rattus rattus*-borne *Leptospira* spp. appeared genetically diverse as *L*. *borgpetersenii*, *L*. *kirschneri* and *L*. *interrogans* were identified for thirteen, 5 and 3 samples, respectively. The sequencing of *rrs2* locus in dog samples identified the infecting *Leptospira spp*. as *L*. *borgpetersenii* (n = 3) and *L*. *kirschneri* (n = 3). We also identified one of the two PCR-positive frugivorous bats as infected by *L*. *kirschneri*. Most importantly, all *Leptospira* strains isolated and/or genotyped from *T*. *ecaudatus* samples were identified as *L*. *mayottensis*.

### Phylogenetic analyses of occurring *Leptospira* spp

**Fig 1:**
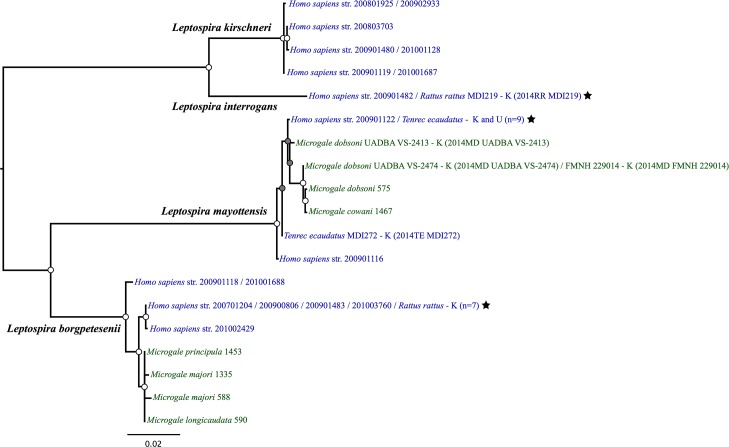
Bayesian phylogenetic tree of pathogenic *Leptospira* species from Mayotte (blue) and Madagascar (green) based on concatenated sequences of five genes (*secY*, *adk*, *lipL32*, *lipL41* and *rrs2*, total size: 2215 bp). The analysis was realized under the GTR+G substitution model. At the nodes, grey and white circles indicate posterior probabilities superior to 0.70 and 0.90, respectively. Strain numbers of cultures produced herein are indicated in parentheses, “K” and “U” designating the sequences obtained from kidney or urine, respectively. Stars indicate lineages common to humans and animals (*Rattus rattus* or *Tenrec ecaudatus*). For *Homo sapiens* str. 200901122, the same sequence type was found in nine cultures from *T*. *ecaudatus* (2014TE MDI222, 2014TE MDI224, 2014TE MDI294U, 2014TE MDI295, 2014TE MDI295U, 2014TE MDI306, 2014TE MD308, 2014TE MDI321 and 2014TE MDI321U). For *H*. *sapiens* str. 200701204, 200900806, 200901483 and 201003760, the same sequence type found was in seven cultures from *R*. *rattus* (2014RR MDI247, 2014RR MDI250, 2014RR MDI251, 2014RR MDI259, 2014RR MDI260, 2014RR MDI284 and 2014RR MDI291). Specimen system: MDI = CRVOI specimen catalogue during field trips to Mayotte; FMNH = Field Museum of Natural History, Chicago; UADBA = Université d’Antananarivo, Département de Biologie Animale, Madagascar; for the other bacterial sequences from *H*. *sapiens* and *Microgale* spp. see Bourhy *et al*. 2012 [15] and Dietrich *et al*. 2014 [4]. Museum numbers for *Microgale* spp.: 575 = UADBA 30869; 588 = UADBA 30289; 590 = UADBA 30291; 1335 = UADBA 32122; 1453 = UADBA 32125; 1467 = UADBA 32101.

We constructed a concatenated phylogeny (2215 bp) by using bacterial sequences from the 16 animal *Leptospira* isolates from Mayotte of the present study, 17 previously described clinical isolates from Mayotte [20] and nine *Leptospira* strains infecting Malagasy endemic Tenrecidae (six previously published [4] and three obtained from three distinct animals sampled in Réserve Spéciale d’Ambohitantely). The phylogeny presented on Fig 1 shows that all bacterial sequences obtained from *T*. *ecaudatus* clustered into the monophyletic *L*. *mayottensis* clade that includes two clinical *L*. *mayottensis* isolates (str. 200901116 and str. 200901122). This clade also contains *Leptospira* sequence types (STs) obtained from *Microgale cowani* and *M*. *dobsoni*, these latter species being tenrecids endemic to Madagascar. Out of the eight sequences obtained from *T*. *ecaudatus* in Mayotte, seven were identical to each others and similar to clinical *L*. *mayottensis* str. 200901122 (100% of pairwise identity based on 2215 bp) and the last one (MDI272) was closely related to the clinical *L*. *mayottensis* str. 200901116 (99.70% of pairwise identity based on 2215 bp). Of note, the couple of kidney/urine cultures obtained from two *T*. *ecaudatus* (MDI295 and MDI321) yielded identical sequence. *Leptospira mayottensis* infecting Malagasy *Microgale* spp. displayed a 27-fold higher nucleotide diversity (π = 0.00298) compared to that infecting *T*. *ecaudatus* from Mayotte in four different sampling sites (π = 0.00011, S1 Fig). The phylogeny confirms *R*. *rattus* as carriers of *L*. *interrogans* and *L*. *borgpetersenii*. For *L*. *interrogans*, the same ST is shared by one *R*. *rattus* (MDI219) and the *L*. *interrogans* clinical isolate str. 200901482 (100% of pairwise identity based on 2215 bp). For *L*. *borgpetersenii*, one clade of this bacterial species included seven sequences obtained from *R*. *rattus* that were almost identical (99.99% pairwise identity, 2215 bp) to five previously described clinical *L*. *borgpetersenii* isolates. Noteworthy, sequences embedded in this clade were clearly distinct from *L*. *borgpetersenii* sequences obtained from Malagasy tenrecids. The nucleotide diversity of *L*. *borgpetersenii* from Malagasy tenrecids (π = 0.00181) was higher than that found in *R*. *rattus* from Mayotte (π = 0, one single ST).

Although full MLST was not successful for bats, dogs and for some of the *R*. *rattus*, we still used *rrs2* sequences alone in order to disentangle the role of these animals in human leptospirosis on Mayotte. For instance, the single leptospiral *rrs2* (obtained using MLST scheme primers) sequence obtained from a frugivorous bat (*P*. *seychellensis comorensis*) was genetically related to clinical *L*. *kirschneri* isolates (98.70% pairwise identity, based on 452 bp) although nucleotide divergence at this locus together with PCR failure on all other loci suggested that *L*. *kirschneri* detected in frugivorous bats was actually divergent from clinical samples (see S2 Fig). Sequences obtained from dogs using the same primers revealed perfect identity with *L*. *borgpetersenii* (n = 3) and *L*. *kirschneri* (n = 2) clinical isolates from Mayotte. Lastly, as these *rrs2* primers failed to produce amplification on some rat samples, we used an alternative primer set (LA-LB) that allowed amplification of a shorter *rrs2* sequence, revealing five identical *L*. *kirschneri* sequences that, importantly, diverged from clinical isolates (3 mismatches out of 245 bp), thus in favour of a predominant role of dogs in *L*. *kirschneri* transmission to humans.

## Discussion

Recent reports of human leptospirosis in the SWIO have stressed specificities singularizing Mayotte from the other islands of the region. *Leptospira* spp. infecting humans on Mayotte are diverse and belong to four bacterial species of which one, *Leptospira mayottensis*, was recently elevated to the rank of new species [9]. This situation clearly contrasts from that occurring on Reunion Island where *Leptospira interrogans* and *Leptospira borgpetersenii* are the only two species reported in human cases [8,21].

Our screening of Mayotte fauna allowed identifying 36 *Leptospira* strains as *L*. *interrogans*, *L*. *borgpetersenii*, *L*. *mayottensis* and *L*. *kirschneri*. We revealed either perfect or nearly perfect identity between (i) the single *L*. *interrogans* ST obtained from patients and *Rattus rattus* (100% identity), (ii) the prevailing *L*. *borgpetersenii* ST obtained from patients and *R*. *rattus* (100% identity), and (iii) both *L*. *mayottensis* STs obtained from patients and *Tenrec ecaudatus* (100% identity and 99.7% identity). The sequencing of *rrs2* locus suggests that *L*. *kirschneri* reported in clinical cases originates from dogs although this phylogeny, based on the single *rrs2* locus, known to display low polymorphism [22], is clearly weaker than the phylogeny based on full MLST. Finally, although a previous study reported a seroprevalence of 10% in *Pteropus seychellensis comorensis* in Mayotte [7], here all urine samples from bats tested negative for *Leptospira* sp. Although we cannot exclude that we missed positive samples due to our limited number of samples and the dynamic nature of leptospires excretion in bats over time [23], the single *L*. *kirschneri rrs2* sequence obtained from a *P*. *seychellensis comorensis* specimen was clearly distinct from the lineages involved in human disease, suggesting that local bat species do not appear to play a significant epidemiological role in the transmission of leptospirosis. Considering bats being the only indigenous mammal species occurring on Mayotte, and dogs, *R*. *rattus* and *T*. *ecaudatus* the more widely represented mammal species in the environment [7], these results highlight the role of introduced wild and domestic small mammal species in the epidemiology of leptospirosis on Mayotte, and are specifically discussed hereafter.

Firstly and most importantly, our results suggest that *T*. *ecaudatus* is a main reservoir of *L*. *mayottensis* as this species was detected only in this host (infection rate: 27.0%) and not in the 289 *R*. *rattus* investigated herein. Although a previous study reported the detection of *L*. *mayottensis* in two out of 20 positive *R*. *rattus* [7], our results on a much larger sampling suggests that this species is not a significant reservoir of *L*. *mayottensis*. Moreover, all *Leptospira* strains infecting *T*. *ecaudatus* on Mayotte were strictly typed as *L*. *mayottensis*, although a larger sample size would be needed to confirm that *L*. *mayottensis* is the only *Leptospira* species infecting *T*. *ecaudatus*.

Secondly, the genetic analyses suggest that *L*. *mayottensis* likely originates from neighbouring Madagascar. Indeed, all *L*. *mayottensis* sequences cluster into a monophyletic well-supported clade including sequences obtained from three terrestrial wild small mammal species, namely *T*. *ecaudatus*, *Microgale dobsoni* and *Microgale cowani*, all belonging to the highly diversified Tenrecidae family representing an adaptive radiation of at least 32 endemic species, which colonized Madagascar some 25–30 million years ago [24]. This introduction scenario is further supported by the narrow genetic diversity of *L*. *mayottensis* in *T*. *ecaudatus* from Mayotte, based on positive samples from four different geographical sites. However, additional sampling of *T*. *ecaudatus* in Madagascar is necessary to confirm this hypothesis.

Thirdly, *R*. *rattus* is a major reservoir of pathogenic leptospires on Mayotte as also reported worldwide. Unexpectedly, the screening of a large number of rat samples (n = 289) from different localities (n = 8) shows that *R*. *rattus* on Mayotte is only marginally infected by *L*. *interrogans*. This feature contrasts with many investigations reported worldwide, including on the neighbouring islands of Madagascar and Reunion Island where rats are only infected with *L*. *interrogans* [8,25]. This peculiarity of Mayotte supports a massive contamination of the environment on this island by a diversity of *Leptospira* species, notably *L*. *borgpetersenii* detected in 62.0% of analysed *R*. *rattus*. Noteworthy, all *L*. *borgpetersenii* and *L*. *interrogans* obtained from *R*. *rattus*, showed perfect identity with human isolates based on the multilocus analysis. Although we identified *L*. *kirschneri* in five *R*. *rattus*, sequence analyses (*rrs2*) are rather supportive of a predominant role of dogs as reservoirs of *L*. *kirschneri* lineages of local medical importance. However, the absence of culture did not allow full genotyping of dog-hosted leptospires and their implication in human transmission needs further investigations.

Fourthly, *L*. *borgpetersenii* STs isolated from acute human cases on Mayotte group with two clearly distinct clades. The first lineage can be traced to *R*. *rattus* and is interestingly also closely related to a clade previously described as associated with endemic Malagasy *Microgale* species [4], indicating a possible host shift from endemic (Tenrecidae) to introduced (rats) mammals. The second *L*. *borgpetersenii* lineage, represented by two human isolates (str. 200901118 and 201001688), appears related to *L*. *borgpetersenii* lineages reported worldwide, e.g. Denmark, China, and Slovakia [4], but not in any wild and domestic animals sampled herein. This cosmopolite lineage may have been introduced through a reservoir, which is yet to be identified.

Finally, the remarkably high success rate to grow in culture *L*. *mayottensis* from *T*. *ecaudatus* (8/10, 80.0%) and *L*. *borgpetersenii* from *R*. *rattus* (7/13, 53.8%) indicates some specific biological peculiarities of these strains on Mayotte. Indeed, using the same protocol, we could grow in culture on Madagascar only three strains of *L*. *mayottensis* (obtained from kidney extracts from *M*. *dobsoni*, included on Fig 1), while other *Leptospira* species were found in terrestrial Malagasy wild animals [4,25]. Hence, this property to readily grow on liquid culture medium may reflect some adaptive advantage to survive and/or multiply in the environment. Interestingly, *L*. *mayottensis* was reported to grow over a wide range of temperature including 11°C and 37°C in contrast to other pathogenic strains [9].

Islands are considered as exceptionally prone to the successful introduction and subsequent invasive nature of exotic animals and plants [26] and have been studied in detail because of adaptive features of species in insular contexts. Our data document that a pathogen most certainly originally endemic to Madagascar [4] has been introduced to the small island of Mayotte where it expanded to become a significant emerging human pathogen. From an evolutionary perspective, our study highlights that beside macro-organism diversity, the associated micro-biodiversity, including endemic microbial lineages, deserves to be included in studies of invasive biology in the context of island biogeography. For instance, although we cannot exclude that other animal species may excrete *L*. *mayottensis* on Mayotte Island, the absence of *L*. *mayottensis* in other animal species together with the monophyly of *L*. *mayottensis* clade, composed of lineages strictly infeoded to tenrecids, support that this new *Leptospira* species has actually coevolved during the Malagasy radiation of tenrecs, as recently proposed for bat-borne *Leptospira* lineages in Madagascar [22].

## Supporting Information

S1 Fig Distribution of small mammals sampling sites on Mayotte. Numbers correspond to the 18 sampling sites where *Rattus rattus* and *Tenrec ecaudatus* (white) and bats (orange) were trapped. Map was created with QGIS 2.8.1 (QGIS Development Team, 2016, QGIS Geographic Information System, Open Source Geospatial Foundation Project). Photography of Mayotte: BD Topo IGN, 2008.
(TIFF)

S2 Fig **The figures A and B display Bayesian phylogenetic trees of pathogenic *Leptospira* from Mayotte (blue) and Madagascar (green) based on 452 bp (57 taxa, HKY+I+G) (A) and 245 bp (64 taxa, K80+I) (B) of the *rrs2* gene.** At the nodes, the black numbers indicate posterior probabilities. The sequences highlighted in grey (**A**) and red (**B**) represent PCR positive samples for which only the *rrs2* gene was obtained. Strain numbers of cultures produced herein are indicated in parentheses, “K” and “U” designating sequences obtained from kidney or urine, respectively. Specimen system: MDI and MAY = CRVOI specimen catalogue during field trips to Mayotte; all *Canis lupus familiaris* were sampled during field trips to Mayotte; FMNH = Field Museum of Natural History, Chicago; UADBA = Université d’Antananarivo, Département de Biologie Animale, Madagascar; for the other bacterial sequences from *Homo sapiens* and *Microgale* spp. see Bourhy *et al*. 2012 [15]and Dietrich *et al*. 2014 [4]. Museum numbers for *Microgale* spp.: 575 = UADBA 30869; 588 = UADBA 30289; 590 = UADBA 30291; 1335 = UADBA 32122; 1453 = UADBA 32125; 1467 = UADBA 32101.
(TIFF)
