# Supplementary figures and images for: Identification of Tenrec ecaudatus, a Wild Mammal Introduced to Mayotte Island, as a Reservoir of the Newly Identified Human Pathogenic Leptospira mayottensis
Source: PLoS Negl Trop Dis. 2016 Aug 30;10(8):e0004933. doi: 10.1371/journal.pntd.0004933 (PMC5004980; doi:10.1371/journal.pntd.0004933)

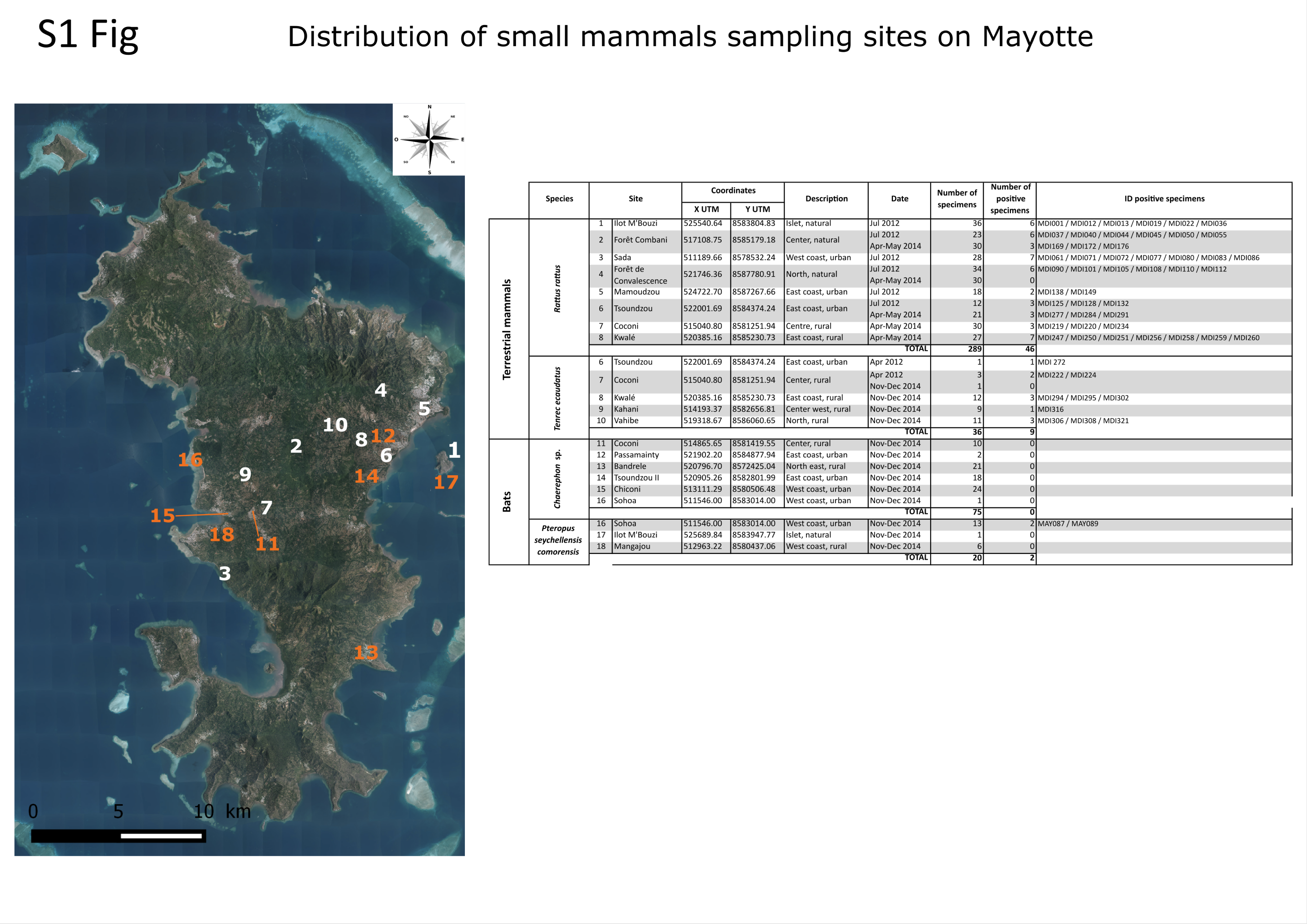

Supplement: S1 Fig — Numbers correspond to the 18 sampling sites where Rattus rattus and Tenrec ecaudatus (white) and bats (orange) were trapped. Map was created with QGIS 2.8.1 (QGIS Development Team, 2016, QGIS Geographic Information System, Open Source Geospatial Foundation Project). Photography of Mayotte: BD Topo IGN, 2008. (TIFF) [file pntd.0004933.s001.tiff]

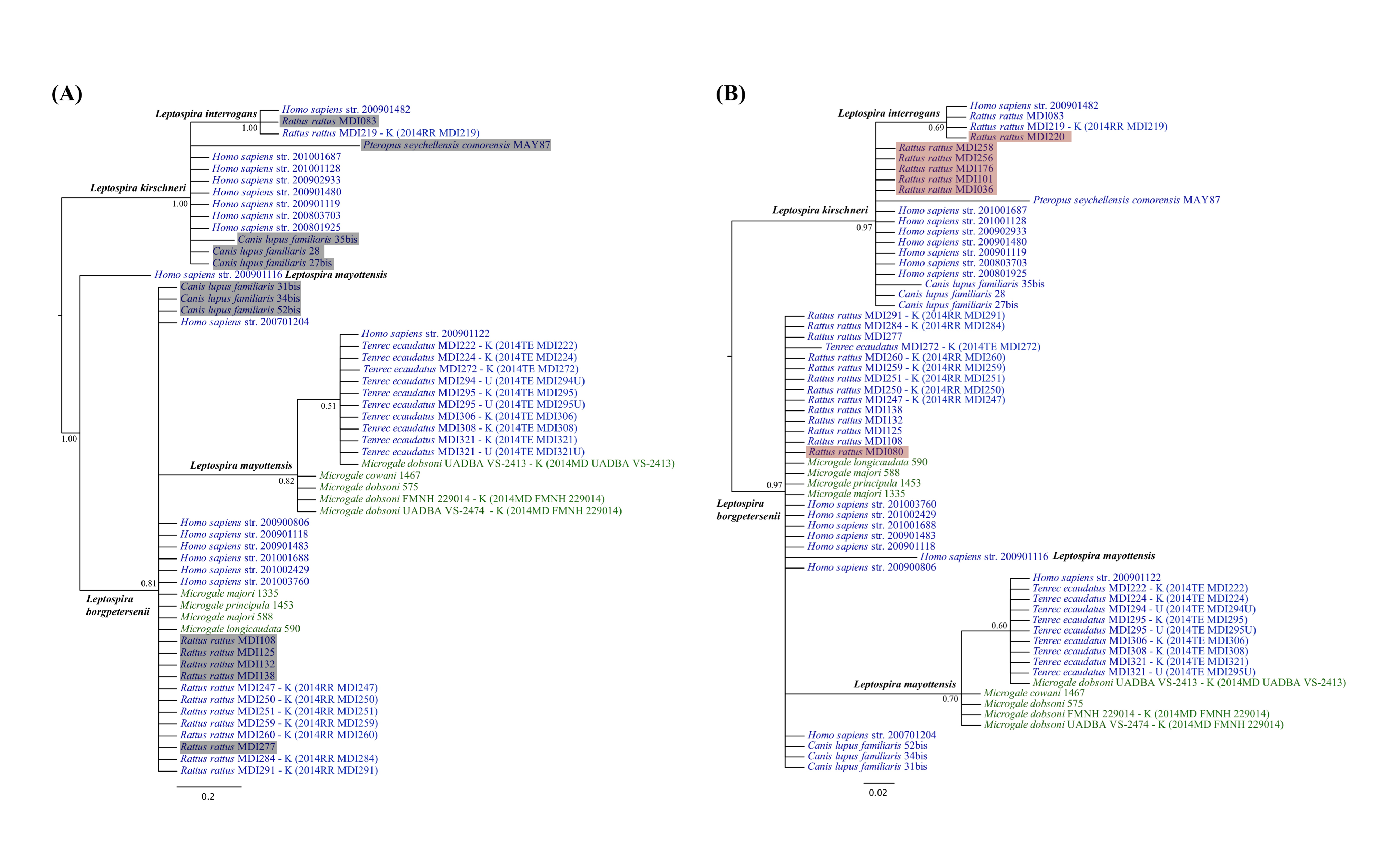

Supplement: S2 Fig — The figures A and B display Bayesian phylogenetic trees of pathogenic Leptospira from Mayotte (blue) and Madagascar (green) based on 452 bp (57 taxa, HKY+I+G) (A) and 245 bp (64 taxa, K80+I) (B) of the rrs2 gene. At the nodes, the black numbers indicate posterior probabilities. The sequences highlighted in grey (A) and red (B) represent PCR positive samples for which only the rrs2 gene was obtained. Strain numbers of cultures produced herein are indicated in parentheses, “K” and “U” designating sequences obtained from kidney or urine, respectively. Specimen system: MDI and MAY = CRVOI specimen catalogue during field trips to Mayotte; all Canis lupus familiaris were sampled during field trips to Mayotte; FMNH = Field Museum of Natural History, Chicago; UADBA = Université d’Antananarivo, Département de Biologie Animale, Madagascar; for the other bacterial sequences from Homo sapiens and Microgale spp. see Bourhy et al. 2012 [15]and Dietrich et al. 2014 [4]. Museum numbers for Microgale spp.: 575 = UADBA 30869; 588 = UADBA 30289; 590 = UADBA 30291; 1335 = UADBA 32122; 1453 = UADBA 32125; 1467 = UADBA 32101. (TIFF) [file pntd.0004933.s002.tiff]
